# Suffering London

**Published:** 1892-06-04

**Authors:** Conrad W. Thies


### The Institutional Workshop.

### HOSPITAL OPINION.

[Contributions critical, suggestive, controversial, and technical are invited for this column, which is open to everyone who has anything to say that is of practical value and interest.]

### SUFFERING LONDON.

(By a Hospital Secretary.)

Doubtless the publication of "Suffering London" is an event which has been eagerly anticipated by many who are interested in the welfare of our metropolitan hospitals, and it is possible that some of the expectations which have been raised in respect to the book will hardly be realised.

From a first perusal of the work it is evident that Mr. Hake has the pen of a ready writer, and that he has stated the needs of the hospitals in an original and graphic manner; it is, however, equally evident that he is not very well versed in hospital work and administration, and one is sorely tempted to controvert some of the statements which he makes. It is more than probable that a book dealing with the subject in a more thorough and serious manner would fail to arouse public attention and sympathy, although it would, doubtless, be more acceptable to those who are already interested in this important department of philanthropic work.

It is not my intention on the present occasion to criticise Mr. Hake's statements—some of which are likely to provoke discussion—but I desire rather to draw attention to what I consider is a serious omission in his plea for our hospitals. Statistics are not attractive to the average reader, but it is necessary to resort to them when we wish to ascertain the actual condition of the people, and the absolute necessity which exists for affording medical relief to the sick poor.

In his remarkable work on "The Labour and Life of the People," Mr. Charles Booth has brought to light some remarkable facts which bear directly upon this subject, for he shows that 30 per cent., or nearly one-third of the population of London, are in a state of chronic poverty, that is, are living under a constant struggle to obtain the actual necessaries of life and make both ends meet. These people are in a state of chronic want; the best off of them receiving less than 21s. per week for the maintenance of a family, out of which small income from 4s. to 7s. per week had to be paid for rent. There are in London 150,000 families each of which occupies only one room, and it is estimated that fully 250,000 children are being brought up amidst such unpromising conditions so fruitful of disease.

In the face of such facts as these it is not difficult to account for the large number of persons who attend the out-patient departments of our hospitals, for it is evident that people living under such conditions are a ready prey to sickness, and, when unable to work, their miserable income at once ceases, and they are quite unable to pay for the medical relief which they so greatly need. It must also be borne in mind, in considering this question, that the London hospitals are largely resorted to by the poor of the rural districts around the metropolis, for it is the common practice for clergymen and other philanthropic persons who visit amongst the poor in country districts to send their protéges up to London for medical advice.

Another aspect of the hospital problem may be referred to here. It is estimated that about £6,000,000 is given annually to the various charitable institutions of London, which are dependent upon voluntary contributions, exclusive of the incomes of the endowed institutions. Of this sum over £3,000,000, or fully one-half, is absorbed by missionary, Bible, and kindred societies, while less than £700,000 is given to hospitals, dispensaries, and other institutions for medical relief.

It seems to me that the knowledge of these facts must emphasise the concluding paragraph of Mr. Walter Besant's forceful preface, "We must have hospitals; we must have voluntary hospitals; we must do something of our own free will—a thing that is not a tax—an offering, a tribute, a recognition of Lazarus as our brother. The cause may be pleaded on religious grounds; it is so pleaded, year after year, and not without effect. It may be pleaded on civic or national grounds for the advance of science, the discovery of things preventive and things curative; the stoppage of things contagious and infectious. It may be pleaded also on purely selfish principles, because the selfish Dives is safest in his luxury when the sufferings of the poor are alleviated by his hand out of his plenty. Charity by cheque may be a very poor kind of charity; but the motive concerns the giver; it may be left to him. The cheque may mean brotherly love and pity; it may mean love of science and the advancement of knowledge; it may mean pure selfishness—a sop to the needy—something to keep him quiet. The motive concerns the giver. *But he must give.*"

May 29th, 1892. Conrad W. Thies.

